# Hematological reference intervals among full-term newborns in Ethiopia: a cross-sectional study

**DOI:** 10.1186/s12887-020-02320-5

**Published:** 2020-09-02

**Authors:** Tegenaw Tiruneh, Teklehaimanot Kiros, Sisay Getu

**Affiliations:** College of Health Sciences, Department of Medical Laboratory Science, Debre Tabor University, Debre Tabor, Ethiopia

**Keywords:** Hematological reference interval, newborns, Debre Tabor, Ethiopia

## Abstract

**Background:**

Hematological reference intervals are used for medical decision tools for interpretation of numerical test results. Establishing of hematological interval among newborn babies is very important for the diagnosis of malignancy, anemia, bleeding disorders, and various infections. There are no locally established hematological reference intervals in Ethiopia. Thus, the aim of this study is to establish locally determined hematological reference interval among full-term newborns.

**Methods:**

A cross-sectional study was conducted from May 15 to July 30 2019 among 151 apparently healthy full-term newborns at Gondar University Hospital. About 3 ml of cord blood was obtained for analysis of Hematological parameters and determined by using Sysmex KX-21N (Sysmex Corporation Kobe, Japan) automated analyzer. Median, 2.5th and 97.5th percentile were computed.

**Result:**

Male to female ratio was almost equal. All hematological parameter had no statistically significant difference between males and females. The delivery types were not influenced its hematological values. The reference interval of white blood cells, red blood cells, platelets, hemoglobin, hematocrit, mean cell volume, and mean cell hemoglobin were (7.64–22.16) x10^9^/l, (3.69–5.47)x10^12^/l, (132.74–413.4) x10^9^/l, (13.32–19.64) g/dl and (39.42–58.06)%, (91.6-113.22)fl, and (30.48–38.02 pg), respectively.

**Conclusions:**

All hematological reference intervals were established from full-term newborns at University of Gondar hospital was different from other studies in Nigeria, Iraq, Pakistan, Nepal, Saudi Arabia and Iran. Therefore, own determined reference value is very important for the clinicians to correctly diagnosis the patients at health facility levels.

## Introduction

### Background

Umbilical cord blood (UCB) is the blood remaining after in the excluding of the placenta. It is considered as biological waste products. However, it has many advantages like stem cell transplantation (rich in hematopoietic progenitor and stem cells), Rhesus factor (Rh) blood typing of the newborns, assessment of the neonatal thrombocytopenia or thrombosis, screening and diagnosis of anemia, polycythemia, bacterial sepsis and determination of hematological reference interval (RI) [1–3]. The other main advantage of UCB is to minimize blood draw from very ill newborns that cause increased morbidity and mortality during neonatal life. The UCB is the alternative source to eliminate unwanted, repeated blood drawn for laboratory tests. The collection of UCB is safe for the patients and technically easy as compared to other blood collection sites [4].

The UCB is composed of white blood cells (WBC), red blood cells (RBC), plasma, platelets and is also rich in hematopoietic stem cells, which have immense potential to cure the malignant and genetic disorder. Therefore, establishing of hematological RI among newborns from UCB is very important for neonatologist for the diagnosis RBC disorders (like anemia and polycythemia), hematological malignancy, platelet disorders (like thrombosis and thrombocytopenia), autoimmune disease, certain genetic disorders problems, monitoring of the patients during in a therapeutic regimen and diagnosis of various bacterial infections [5–7]. It is widely known that newborn hematological parameters are different from those of infants or adults. Therefore, separated hematological RI is mandatory for newborns [8].

The RI is the range between values from the lower reference limit to the upper reference limits [9]. They are ideally defined on apparently healthy individuals, and should be distinguished from clinical decision limits that are derived from known diseased patients. It is a distribution of numerical test results expected in a representative population of healthy individuals. It is mainly used to make medical decisions about hematological disease diagnosis, treatment & monitoring of disease prognosis of the patients [10]. According to Clinical Laboratory Standards Institute guideline, the RIs are defined in relation to a healthy population to include the values in which 95% of apparently healthy individuals would fall and in which 2.5% of results in the lower range are out of the RI and 2.5% of values in the upper range will be out of the RI [11, 12].

Hematological RIs are the most common medical decision-supporting tools used for the interpretation of numerical hematological test results. Accurate patient result interpreting within the correct RI is mandatory to minimize the patient risks from disease, increase recovery rates, and improve monitoring therapy of various hematological disorders [13]. Almost 80% of physicians’ medical decisions are based on information provided by laboratory reports. Therefore, locally established hematological RI among newborns is very important for the physician to correctly diagnosis and early treatment of the patients [14].

In Ethiopia there is no locally determined hematological RI among healthy full-term newborns in the country, particularly is in the study area. They used in Western and American RI. Since hematological RI is varying through age, race, ethnicity, altitude, sex, drug intake, time of sampling and socioeconomic status. It is commonly known that newborn hematological parameters are different from those of neonates, infants or adults [8]. It needs locally determined RI. Due to this reason we motivate to conduct this study. The aim of this study was to establish hematological RI by using UCB of apparently healthy, full-term newborns at University of Gondar compressive specialized hospital, northwest Ethiopia.

## Method

### Study Setting, design and population

An institution-based cross-sectional study was conducted from May 15 to July 30 2019, at gynecology and obstetrics department of University of Gondar comprehensive specialized hospital, Northwest Ethiopia. The hospital is located in Gondar town Amhara regional state, northwest Ethiopia. The town is located 2,133 m elevation above sea level. This hospital provides both teaching and referral center in the region, which services more than five million people. Currently, the hospital holds more than 550 beds, and it handles approximately 8000–9000 deliveries per year. The hospital has a range of specialties including pediatrics, surgery, gynecology, psychiatry, human immunodeficiency virus (HIV) care, and an outpatient clinic.

The newborns were selected based on the following criteria: Birth at full-term (39–42 weeks of gestation), and absence of any congenital anomalies. All selected newborns were physically examined at birth and found normal and apparently healthy. The premature newborn (delivered less than 37 weeks of gestation), twin newborns, the pregnancy complicated with diabetics, preeclampsia, hypertension, HIV/AIDS, chronic kidney, liver disease, malaria, anemia, and hematological malignancy were excluded from the study. On the other hand, a mother who had bleeding during pregnancy, maternal drinking of alcohol during pregnancy, cigarette smoking during pregnancy, and no antenatal care also were excluded from being sampled. A systematic random sampling technique was employed to select study participants. The sample size is determined based on the national Committee for Clinical Laboratory Standards, International Federation of Clinical Chemistry and Clinical Laboratory Standards Institute guideline recommendations, a minimum size of 120 observations is required for determination of RIs [15]. Study participants were selected every three intervals based on flow of delivery to avoid bias and equal allocation of study participants. The excluded study participants were substituted with the next consecutive study participants. A total of 202 study participants were selected during the study periods. However, 43 were excluded due to the presence of maternal anemia, 5 were excluded due to twins and the remained 3 newborns were still birth. Finally, a total of 151 newborns with their respective mothers were included and their results were analyzed in SPSS.

### Data collection method

#### Socio-demographic and clinical data collection

A pre-tested structured questionnaire prepared in English and translated to the local language (Amharic) was used to obtain newborn gender, birth weight, and presence of bleeding during pregnancy, alcohol consumption habits, and cigarette smoking during pregnancy via face-to-face interviews. The presence of maternal complications like malignancy, hypertension, diabetics, tuberculosis, HIV/AIDS, chronic kidney, and liver disease were retrieved from maternal medical records with the aid of data extraction sheet.

#### Blood collection and laboratory analysis

About 3 ml of UCB specimen were obtained from each study participants after delivery from the clamped umbilical cord. The two trend Midwifery professionals collected the cord blood sample from the clamped cord through excluding of the placenta. The collected sample was immediately poured into tri-potassium ethylene diamine tetra acetic acid (K3-EDTA) test tube and gently mixed to prevent blood clotting. In addition, 3 ml of venous blood was collected from the mother after delivery with a sterile and disposable syringe. Hematological parameters: total white cell count (WBC), differential white cell count (neutrophils, lymphocytes and mixed which contains eosinophils, monocytes. and basophiles), platelet count, red blood cell count (RBC), Hgb, hematocrit (%), mean cell volume (MCV), mean cell hemoglobin (MCH), mean cell hemoglobin concentration (MCHC), and red cell distribution width (RDW) were determined by using the using Sysmex KX-21N (Sysmex Corporation Kobe, Japan) automated hematological whole blood analyzer based on direct current principle. Three experienced laboratory technologist performs the complete blood count (CBC) by strictly adhering standard operating procedures.

#### Data quality control

In order to increase the reliability of data, training was given to the data collector prior to data collection. During laboratory data collection standard operating procedures were strictly followed and implemented from prior to specimen collection up to recording and interpreting of the laboratory results. The collected sample was immediately dispensed to the wall of the EDTA test tube slowly and properly mixed by inverting the tube gently 8–10 times to prevent hemolysis of blood samples. Then label of the sample and the request paper with the same identification number in order to avoid any mix up of errors. The expired date of the reagent was checked before analysis of patient samples. Daily installations and background run were done to minimize any background errors. Repeated analysis of randomly selected samples of reproducibility check was carried out three times a week to evaluate instrument performance consistently and precisely.

### Statistical analysis

The data were cleaned, edited, checked for completeness, and entered into Epi-info version 7.2.1.0. Then it was exported into SPSS version 20 for analysis. Outliers were detected by using Z score and Tukey method. Before any analysis, normal distribution of numerical data was checked using the Kolmogorov-Smirnov and Shapiro-Wilk test. Since all data are not Gaussian distributed we used non-parametric tests to determine the hematological RI as recommended as the clinical laboratory standard institutes [12]. Median with interquartile range (IQR), and 95% confidence interval was computed. The 97.5th percentile and 2.5th percentile were the upper and the lower reference limit of the study populations, respectively. Mann-Whitney U test also used to test for the mean difference of variance between gender (male versus female), and delivery type (vaginal versus cesarean section). According to the guideline of clinical laboratory standard institutes, we determine the 95% hematological RI by computing 97.5th percentile and 2.5th percentile.

## Results

### Sociodemographic and obstetric data of study participants

A total of 151 healthy newborns (76 females and 75 males) were included in this study. Majority of study participant (87.4%) were born from mother came from urban residence with normal birth weight (90.1%). About 81.5% of study participants were born through spontaneous vaginal delivery route and the remaining were by cesarean sections (Table 1).

**Table 1:** Sociodemographic characteristics of study participants

Variable		Frequency	Percentage (%)
Gender	Male	75	49.7
	Female	76	50.3
Residence of mother	Urban	132	87.4
	Rural	19	12.6
Occupation of the mother	Employed	54	35.8
	House wife	57	64.2
Birth weight	Normal ( > = 2.5 kg)	136	90.1
	Under birth (< 2.5 k.g)	15	9.9
Gravidia	Primigravidia	65	43
	Multigravidia	86	57

The minimum versus maximum value of cord WBC, cord RBC, and cord platelet among study participants were (3.6 vs. 27) x10^6^/L, (3.02 vs. 5.7) x10^12^/L, (132 vs. 500) x10^9^/l, respectively. As a result, summarized in Table 2 sex and delivery type had no influence for cord hematological values (*p*-value > 0.05). The median value of cord WBC, RBC, Hgb, and Hct among male newborns were 12.6, 4.7, 16.2 and 49.6, respectively. On the other hand, the median value WBC, RBC, Hgb, and Hct among female newborns were 13.15, 4.6, 15.75, and 48.65, respectively (Table 2).

**Table 2:** The influence of sex and delivery type on hematological parameter in newborns based on median value

Hematological parameter	Sex			Delivery type		
	Male	Female	*P*-value	Vaginal	Cesarean	*p*-value
WBC(x10^9^/l)	12.6	13.15	0.98	12.6	13.1	0.64
RBC(x10^12^/l)	4.7	4.6	0.51	4.61	4.73	0.27
Hgb (g/dl)	16.2	15.75	0.47	15.8	16.6	0.09
Hct (%)	49.6	48.65	0.44	48.6	50.45	0.05
Platelet (x10^9^/l)	292	271	0.17	276	274	0.62
Lymphocyte absolute (x10^9^/l)	4.5	4.6	0.12	4.6	4.55	0.91
Neutrophil absolute ((x10^9^/l)	6.4	7.25	0.19	7	6.95	0.57

### Hematological reference interval

The newborns hematological RI is determined based on non-parametric estimation methods. Based on the SPSS statistical analysis of newborn laboratory data did not require partition by sex groups. Based on the finding, all hematological value did not have a statistically significant difference of means among sex groups as well as the route of delivery types. According to clinical laboratory approve guideline partitioning was required when the difference between the observed means of two subclasses is statistically significant at the 5% probability level [9]. The median and 95% of RI of RBC, Hgb, Hct, MCV, MCH and MCHC were 4.64 (3.69–5.47 × 10^12^/l), 16 (13.32–19.64), 49.1% (39.42–58.06%), 105.85 fl. (91.6-113.22 fl.), 35 pg (30.48–38.02 pg) and 33.1 g/dl (31.48–36.5 g/dl), respectively. The lower and upper limits WBC and platelets were 7.64, 22.16, and 132.74, 413.4, respectively (Table 3).

**Table 3:** The reference interval and median value of all hematological parameter among full-term newborns (*N* = 151)

Hematological parameter	Median (IQR)	Lower limit (2.5th )	Upper limit (97.5th )	RI (95th percentile)
Total WBC (x10^9^/l)	12.8 (10.5–15.4)	7.64	22.16	7.64–22.16
RBC (x10^12^/l)	4.64 (4.25–4.89)	3.69	5.47	3.69–5.47
Hgb (g/dl)	16 (14.8–17)	13.32	19.64	13.32–19.64
Hct (%)	49.1 (44.9–51.8)	39.42	58.06	39.42–58.06
MCV (fl.)	105.85 (102.6-109.6)	91.6	113.22	91.6-113.22
MCH (pg)	35 (33.7–35.7)	30.48	38.02	30.48–38.02
MCHC (g/dl)	33.1 (32.5–33.5)	31.48	36.5	31.48–36.5
Platelet (x10^9^/l)	276 (233–314)	132.74	413.4	132.74–413.4
Lymphocyte percentage (%)	35.9 (30–41)	14.22	58.32	14.22–58.32
Mix percentage (%)	10.3 (7.9–12.5)	3.34	18.64	3.34–18.64
Neutrophil percentage (%)	53.5 (48.5–60.7)	36.74	79.48	36.74–79.48
Lymphocyte absolute (x10^9^/l)	4.6 (3.6–5.5)	2.16	10.3	2.16–10.3
Mix absolute (x10^9^/l)	1.3 (0.9–1.7)	0.4	2.94	0.4–2.94
Neutrophil absolute (x10^9^/l)	7 (5.4–8.9)	2.96	13.54	2.96–13.54
RDW-SD	68.1 (64.7–72.1)	55.48	81.3	55.48–81.3
Platelet distribution width (PDW)	11.3 (10.5–12.2)	8.8	15.68	8.8-15.68
Mean platelet volume (MPV)	9.5 (9.1–10)	7.88	11.04	7.88–11.04

## Discussion

This study tries to find out the RI among newborns by using UCB which was the first time reported in Ethiopia. The current finding showed that there was no statistically significance difference of all hematological parameters between male and female newborns, which was consistent with a study reported in Sokoto, Northern Nigeria [16] and Lagos, Nigeria [17]. The present study also showed that mode of delivery had no influence of hematological parameters of the newborns which agrees to a study done by Fady M, et al. reported that there was no significant difference in the MCV, MCH, MCHC, RDW, lymphocytes, and monocytes [18]. However, this result is contrasted to a study done by L Glasser et al. showed that mode of delivery had influenced to hematological test values [19]. This variation might be due related to low sample size used in the current study (151 versus 10,287 study participants) as compared to the previous study.

The Hgb values (13.3–19.6 g/dl) of this study almost agrees with a study reported in Saudi Arabia (15.7–19.7 g/dl), however, the upper limit of platelet value (413 × 10^9^/l) the present study was greater than as compared to the upper limit of Saudi reported (297 × 10^9^/l) [20]. On the other hand, Our finding of the 95% RI of RBC (3.69–5.47) values was comparable to a study reported in Nepal (3.67–4.93) [21], Iran (3.61–5.29) [22], and Nigeria (3.52–4.62) [23] reported. Similarly, the 95% RI of WBC (7.64–22.16 × 10^9^/l) in our study similar to the study conducted in Turkey (71.90–25.44 × 10^9^/l) [24].

The lower limit WBC (7.64) value of this study was lower than a study reported in Saudi Arabia (10.9) [20], Nepal (10.49) [21] and Pakistan (9.7) [25]. On the contrary, the lower limit of WBC was higher than a study reported in Sagamu, Nigeria (4.2) [23] and Iran (5.16) [22]. However, the MCH RI (30.5-38.02) were similar to a study done in Saudi Arabia (30.2–40.6) [20], Nepal (31.6–36.2), [21], and Pakistan (32.2–35.4) [25]. On the other hand, the Hct value (39.4–58.1) of the current study was comparable to a study conducted in Iran (39.6–56.9) [22]. This may be due to the variation in geographical location, and race or genetic factors may be contributing to the presence of this difference.

The lower limit of RBC (3.69 × 10^9^/l) in this finding was lower than a study conducted in Saudi Arabia (5.1 × 10^9^/l) [20] and Sagamu Nigeria (4.3 × 10^9^/l). On the contrary, the higher limit of RI of Hgb value (19.6 g/dl) of this study was consistent to a study reported in Saudi Arabia (19.7 g/dl) [20] but higher than in a study reported in Logas Nigeria (14.8 g/dl) [17], Pakistan (17.3 g/dl) [25], Iraq (15.22 g/dl) [26], and Nepal (17.2 g/dl) [21]. The difference of Hgb value might be due to method variation, inclusion criteria of the study participants and time of cord clamping. Similarly, the upper limit of both WBC and platelet values was higher than in the study conducted in Nepal, Pakistan, Logas Nigeria, Iran and Iraq [17, 21, 22, 25, 26] (Table 4).

**Table 4:** Comparison of the current study to other similar findings based on cord blood hematological parameters

Hematological parameter	RI							
	Northwest Ethiopia (*N* = 151)	Saudi Arabia (*N* = 2163) [20]	Nepal (*N* = 210) [21]	Pakistan (*N* = 316) [25]	Lagos, Nigeria (*N* = 130 [17]	Mashhad, Iran (*N* = 447) [22]	Iraq (*N* = 220) [26]	Sagamu, Nigeria (*N* = 108) [23]
Total WBC	7.64–22.16	10.9–21.5	10.49–19.4	9.7–17.7	7.9–18.3	5.16–18.2	7.32–12.92	4.2–25.8
RBC	3.69–5.47	5.1–16.3	3.67–4.93	-	3.52–4.62	3.61–5.29	3.53–4.47	4.3–5.9
Hgb	13.3–19.6	15.7–19.7	13.28–17.2	13.5–17.3	11.8–14.8	13-18.8	12.3-15.22	13-17.8
Hct	39.4–58.1	46.9–59.5	-	-	39-50.6	39.6–56.9	39.7–49.2	39.1–53.5
MCV	91.6-113.2	97.9-112.3	95.2-107.2	103.8–108	98.5-122.2	97.5-119.8	105.5-117.7	84.4–98.8
MCH	30.5-38.02	30.2–40.6	31.6–36.2	32.2–35.4	28.5–36.7	31.7–40	32.1–36.8	27.9–33.1
MCHC	31.48–36.5	27-40.2	31.7–34.9	-	28.1–31.4	30.1–35.2	29.03–32.86	31.7–34.5
Platelet	132.7–413	178.2–297	165.6-288.2	223–347	152.9-297.3	131–383	207–328	129–607
Lymphocyte %	14.2–58.3	16.5–37.1	23.7–46.7	-	-	-	29.6-49.98	27.8–49.6
Neutrophil percentage	36.7–79.5	50.3–74.1	51.5–74.7	-	-	-	39.8–62.2	45.9–67.7
Lymphocyte absolute	2.16–10.3	-	-	3.3–6.9	-	1.14–8.46	-	-
Neutrophil absolute	2.96–13.54	-	-	4.7–10.7	-	1.12–10.2	-	-

*N* =sample size

The platelet counts of this study range from (132.7–413) x10^9^/L. Based on our finding less than 132 × 10^9^/L is thrombocytopenia and greater than 413 × 10^9^/L is thrombocytosis for newborns. The lower limit of platelet counts in the current study (132.7 × 10^9^/L) was consistent to a study reported from Mashhad, Iran (131 × 10^9^/L) [22]. However, the higher limit of platelet value (413 × 10^9^/L) of the current study was higher a study reported by Marwaha et al. (142 × 10^9^/L) [27]. The major limitation of this study was nucleated RBC is not corrected when the total WBC was counted at the time of analysis and use of small sample size.

## Conclusions

There was no statistical significance of RI of all hematological value between male and female newborns. The Hgb values of this study almost agrees with a study reported in Saudi Arabia. The RI of RBC of the current study was comparable to Nepal, Iraq and Nigeria findings. However, the lower limit of WBC was lower than a study reported in Saudi Arabia, Nepal, and Pakistan. The current finding of RI is very helpful for the neonatologist to accurately diagnose and monitoring of therapies of the patients.

## Data Availability

The datasets used and/or analyzed during the current study available from the corresponding author on reasonable request.

## Abbreviations

CBC: Complete Blood Count
Hct: Hematocrit
Hgb: Hemoglobin
MCH: Mean Cell Hemoglobin
MCHC: Mean Cell Hemoglobin Concentration
MCV: Mean Cell Volume
MPV: Mean platelet volume
PDW: Platelet distribution width
RBC: Red Blood Cell
RDW: Red Cell Distribution Width
Rh: Rhesus factor
RI: Reference Interval
WBC: White Blood Cell

## References

[1] Katsares V, Paparidis Z, Nikolaidou E, Karvounidou I, Ardelean K-A, Drossas N (2009). Reference ranges for umbilical cord blood hematological values. Laboratory Medicine.

[2] Wiedmeier SE, Henry E, Sola-Visner M, Christensen R (2009). Platelet reference ranges for neonates, defined using data from over 47 000 patients in a multihospital healthcare system. Journal of perinatology.

[3] Wall D (2011). Umbilical cord blood: importance of supporting public banks.

[4] Carroll P, Nankervis C, Iams J, Kelleher K (2012). Umbilical cord blood as a replacement source for admission complete blood count in premature infants. J Perinatol.

[5] Slhessarenko N, Andriolo A (2016). The importance of determining reference intervals for Laboratory Medicine. Jornal Brasileiro de Patologia e Medicina Laboratorial.

[6] Zech NH, Broer N, Ribitsch I, Zech MH, Broer K-h, Ertan K (2010). The rationale behind collecting umbilical cord blood. Journal of the Turkish German Gynecological Association.

[7] Xanthou M (1970). Leucocyte blood picture in healthy full-term and premature babies during neonatal period. Arch Dis Child.

[8] Noguera NI, Detarsio G, Perez SM, Bragos IM, Lanza O, Rodriguez JH (1999). Hematologic study of newborn umbilical cord blood. MEDICINA-BUENOS AIRES-.

[9] Sasse EA (2000). How to define and determine reference intervals in the clinical laboratory; approved guideline. NCCLS documents C28-A2.

[10] Friedrichs KR, Harr KE, Freeman KP, Szladovits B, Walton RM, Barnhart KF (2012). ASVCP reference interval guidelines: determination of de novo reference intervals in veterinary species and other related topics. Veterinary clinical pathology.

[11] Castellone D (2017). Establishing reference intervals in the coagulation laboratory. International journal of laboratory hematology.

[12] Horowitz G, Altaie S, Boyd J, Ceriotti F, Garg U, Horn P. Defining, establishing, and verifying reference intervals in the clinical laboratory; approved guideline, CLSI document C28-A3. *Clinical and Laboratory Standards Institute, Wayne, PA*. 2008.

[13] Jones G, Barker A (2008). Reference intervals. The Clinical Biochemist Reviews.

[14] Katayev A, Balciza C, Seccombe DW (2010). Establishing reference intervals for clinical laboratory test results: is there a better way?. Am J Clin Pathol.

[15] Sasse Edward A, Doumas Basil T, D’Orazio Paul EJH, Evans Susan A, Graham Gary A, Myers Gary L, et al. Stanton Noel v: How to Define and Determine Reference Intervals in the Clinical Laboratory Approved Guideline. *NCCLS Document C28-A2 2nd edn National Committee for Clinical Laboratory Standards*.

[16] Imoru M, Momodu I, Buhari H, Nuhu A (2016). Haematological Reference Values for Full Term Healthy Neonates Delivered Within 24 Hours in Sokoto, Northern Nigeria. International Journal of Hematology Therapy.

[17] Adewumi A, Adeyemo TA, Akinsegun AA, Abidoye G, Ebele U, Sulaimon AA. Cord blood full blood count parameters in Lagos, Nigeria. Pan Afr Med J. 2014;17(1) 1. 10.11604/pamj.2014.17.192.3680 PMC4228999 25396018

[18] El Gendy FM, Allam AA, Allam MM, Allam RK (2016). Haematological parameters of newborns delivered vaginally versus caesarean section. Menoufia Medical Journal.

[19] Glasser L, Sutton N, Schmeling M, Machan J (2015). A comprehensive study of umbilical cord blood cell developmental changes and reference ranges by gestation, gender and mode of delivery. J Perinatol.

[20] Alharbi S, Alkhotani A (2017). Hematological reference values for full-term, healthy newborns of Jeddah, Saudi Arabia. Journal of Clinical Neonatology.

[21] Basnet S, Singh SK, Sathian B, Mishra R (2016). Reference Ranges for Hematological Values in Umbilical Cord Blood in Pokhara, Nepal. Journal of Nepal Paediatric Society.

[22] Keramati MR, Mohammadzadeh A, Farhat AS, Sadeghi R (2011). Determination of Hematologic Reference Values of Neonates in Mashhad-Iran. International Journal of Hematology Oncology.

[23] Ogundeyi M, Olarewaju D, Njokanma O, Ogunlesi T (2011). Haematological profile of apparently healthy term babies aged one day, three days and six weeks delivered in Sagamu, Nigeria. Nigerian Journal of Paediatrics.

[24] Özyürek E, Cetintaş S, Ceylan T, ÖğÜş E, Haberal A, Gürakan B (2006). Complete blood count parameters for healthy, small-for‐gestational‐age, full‐term newborns. Clinical Laboratory Haematology.

[25] Pasha W, Ali W, Ahmed N, Khattak AL, Idris M, Nayyer ZA (2015). Reference haematological values for full term healthy newborns from rural Sindh, Pakistan. Journal of Ayub Medical College Abbottabad.

[26] Al-Marzoki JM, Al-Maaroof ZW, Kadhum AH (2012). Determination of reference ranges for full blood count parameters in neonatal cord plasma in Hilla, Babil, Iraq. Journal of blood medicine.

[27] Marwaha R, Narang A, Thusu K, Garewal G, Bhakoo O (1992). Routine hematological values in term newborns. Hemoglobin.

